# Immunosenescence and Skin: A State of Art of Its Etiopathogenetic Role and Crucial Watershed for Systemic Implications

**DOI:** 10.3390/ijms24097956

**Published:** 2023-04-27

**Authors:** Vincenzo Papa, Federica Li Pomi, Francesco Borgia, Mario Vaccaro, Giovanni Pioggia, Sebastiano Gangemi

**Affiliations:** 1 Department of Clinical and Experimental Medicine, School and Operative Unit of Allergy and Clinical Immunology, University of Messina, 98125 Messina, Italy; papavi.994@gmail.com (V.P.); sebastiano.gangemi@unime.it (S.G.); 2 Section of Dermatology, Department of Clinical and Experimental Medicine, University of Messina, 98125 Messina, Italy; federicalipomi@hotmail.it (F.L.P.); mario.vaccaro@unime.it (M.V.); 3 Institute for Biomedical Research and Innovation (IRIB), National Research Council of Italy (CNR), 98164 Messina, Italy; giovanni.pioggia@cnr.it

**Keywords:** immunosenescence, inflammaging, skin, melanoma, squamous cell carcinoma, atopic dermatitis, psoriasis, skin infectious disease, cytokine network

## Abstract

Immunosenescence is a complex multifactorial phenomenon consisting of wide-ranging remodeling of the immune system during the life span, resulting in an age-related qualitative–quantitative decline of immune cells and cytokines. A growing body of evidence in the international literature is highlighting the etiopathogenetic role of skin immunosenescence in the onset of various dermatologic conditions. Skin immunosenescence also serves as an interesting watershed for the onset of system-wide conditions in the context of allergic inflammation. Moreover, in recent years, an increasingly emerging and fascinating etiopathogenetic parallelism has been observed between some mechanisms of immunosenescence, both at cutaneous and systemic sites. This would help to explain the occurrence of apparently unconnected comorbidities. Throughout our review, we aim to shed light on emerging immunosenescent mechanisms shared between dermatologic disorders and other organ-specific diseases in the context of a more extensive discussion on the etiopathogenetic role of skin immunosenescence. A promising future perspective would be to focus on better understanding the mutual influence between skin and host immunity, as well as the influence of high inter-individual variability on immunosenescence/inflammaging. This can lead to a more comprehensive “immunobiographic” definition of each individual.

## 1. Introduction

### 1.1. Immunosenescence: From a Common Definition to a More Specific One

The commonly accepted definition of immunosenescence refers to a complex and multifactorial phenomenon that involves a wide-ranging remodeling of the immune system throughout the lifespan. This results in an age-related qualitative–quantitative decline of immune cells and cytokines. The clinical implications of this immunological plasticity are the onset of a wide range of age-related diseases, including metabolic, cardiovascular, and neurodegenerative disorders, cancer, autoimmune diseases, and the emerging COVID-19 [1,2]. In this scenario, an emerging causal role would be attributable to the greater representativeness in the elderly of the immunomodulatory function of the peripheral double-negative T lymphocyte subpopulation, resulting in the suppression of simple-positive T cells [3]. In addition to the general notion of immunosenescence, ongoing studies aim to provide a more specific definition of immunosenescence as a state characterized by significant differences in the expression of immunological biomarkers between younger and older individuals, which is closely correlated with unfavorable clinical outcomes [4]. Interestingly, this definition may also extend across non-human species with mirrored effects, as demonstrated by the first study on age-related changes in avian immunity [5]. 

### 1.2. Skin Immunosenescence: Structural and Functional Hallmarks 

The pathophysiological backbone of skin immunosenescence is characterized by the interaction of two complementary processes at both molecular and cellular levels. The first process is the direct impact of immune cell aging and its related events, including pro-inflammatory cytokine habitus, genomic instability, changes in cellular metabolic pathways, phagocytic hypo/a-responsiveness, and impaired vaccine response. The second process is the indirect influence of cellular aging in the tissue, resulting in non-specific [6] and specific immunological patterns of molecular signaling in response to the weakening of various anatomical barriers. This acts as an additional trigger for functional decay [7]. In this dynamic scenario, the three domains of the immune system are affected. The first domain is the protective epithelial skin and mucous membrane barrier, which acts as the body’s first line of defense. The second domain is the second barrier of innate immunity [8], which has altered allocation and functionality in certain cellular constituents [9]. It also has an age-related low rate of chronic inflammation and a poor response to pathogens. The third domain is the barrier of acquired immunity, which has age-related reduced hematopoiesis of naïve lymphocytes [8]. Furthermore, functional changes related to skin immunosenescence include reduced delayed skin hypersensitivity [10]. This reduction is plausibly linked to an aging-related maturative inhibition of dendritic cells and a functional decline in Th1 immunity. This decline is secondary to the establishment of a redox imbalance due to excessive reactive oxygen species (ROS) production mediated by immune cells [11]. The production of ROS can also be triggered by dietary factors, such as macrophage production of ROS induced by polyunsaturated fatty acids. Subsequent pro-inflammatory effects are specifically demonstrated for omega-3 fatty acid-induced ROS/IL-36 signaling [12,13]. This functional impairment would be reversible with the restoration of glutathione [14]. These outcomes have prompted the most recent attempts to emulate the impairment of the human delayed-type hypersensitivity response in the mouse model, aiming to develop interventions to improve skin immunity in the elderly [15]. An increasingly detailed understanding of mechanisms underlying immunosenescence is leading to the development of anti-immunosenescence and anti-inflammaging strategies. These include adopting a healthy diet, engaging in regular exercise, getting restorative sleep, avoiding stress, staying hydrated, using probiotics, receiving regular vaccine boosters, and considering the therapeutic potential of hormonal treatments and vitamin D supplementation [16,17,18]. From what has been discussed so far, the skin represents the first line of defense against external insults with its structural and immune cells playing crucial roles. The structural cells include keratinocytes, dermal fibroblasts, and adipocytes, while immune cells include macrophages, dermal dendritic cells, Langerhans cells (LCs), T resident memory cells, forkhead box P3+ (FOXP3+) T regulatory (Treg) cells, and mast cells. Therefore, the skin is an interesting watershed for immunosenescence and its system-wide implications. Chambers et al. pointed out that the hallmarks of cutaneous immunosenescence are represented by the numerical and migratory capacity decline of LCs in addition to their hypoexpression of antimicrobial peptides. Moreover, cutaneous immunosenescence is also influenced by structural decay, which is mainly represented by keratinocyte atrophy, downsizing of fibroblasts, and a consequent reduction in the total amount of collagen. This decay also leads to increased fragmentation targeted by matrix metalloproteinases (MMPs) [19]. The impaired migratory and phagocytic capacity of dermal dendritic cells, along with their poor T-cell activating capability, also play a crucial role. In the context of adaptive immunity decline, immunosenescence is marked by the exhaustion and functional inhibition of effector T cells, primarily through programmed cell death protein 1 (PD-1) signaling. This is accompanied by an increase in the number of FOXP3+ Treg cells, which are part of an immune control mechanism for chronic low-grade systemic inflammation, known as “inflammaging.” This chronic inflammation is sustained by aged T lymphocytes, which undergo specific qualitative and quantitative rearrangements in a hierarchically interlinked manner [20].

### 1.3. SASP and SAASP, Two Sides of the Same Coin: Inflammaging

The proinflammatory cytokine release as part of the senescence-associated secretory phenotype (SASP) of senescent cells is predominantly responsible for inflammaging [21]. However, age-related interactions between the skin microbiome and host immunity also contribute to the inflammaging/immunosenescence phenotype. This interaction has high inter-individual variability, resulting in the establishment of a strictly personal immunobiography phenotype, labeled “immunobiography” [22]. Due to the preponderant role of skin senescent cells in the establishment of this inflammatory process, especially referring to the driving force of macrophages, more recently the term “skin aging-associated secretory phenotype” (SAASP) has been coined [23,24,25]. Within this novel phenotype, which is mainly represented by senescent fibroblasts, there is an overlap of components between the SASP and the SAASP. These components include increased levels of matrix MMPs-1, -3, -10, and -14, interleukin (IL)-8, IL-1b, and interferon (IFN)-γ. Additionally, about 30 new junctional pathway-related proteins have been identified as being exclusive to the SAASP phenotype. In contrast, senescent keratinocytes have a limited effect on the SAASP phenotype, with the exception of increased release of IL-1a and the overexpression of plasma-binding proteins, such as ORM1. These proteins are involved in the transportation of circulating inflammatory mediators, which could have systemic inflammatory implications starting from the skin. Among the skin-resident immune cells, effector T cells contribute to the SAASP phenotype through the upregulation of tumor necrosis factor (TNF)-α and IFN-γ along with a CD4+ T cell-related pro-inflammatory T helper (Th)17 phenotype [25]. 

Building upon the review by Dinarello et al., a potentially significant role in the SAASP phenotype and its unknown systemic effects could be attributed to IL-18. The precursor of this cytokine is expressed by keratinocytes as well as most epithelial cells [26]. The potential aging-related overexpression of IL-18 could lead to the promotion of Th2-mediated diseases and autoimmune diseases through pathways involving the upregulation of vascular cell adhesion protein 1 (VCAM-1) and T cell-mediated IL-17. On the other hand, an aging-related downregulation of another important cytokine, IL-27, could have an impact on the skin by reducing the gene expression of IL-18 binding protein (IL-18BP), which is a high-affinity protein that binds to IL-18 and has several anti-IL-18 effects [26]. Given the evidence of a strong increment in both circulating IL-18 and IL-18BP in healthy centenarians, it can be presumed that age-related immune mechanisms in the skin contribute to both IL-18 overexpression and the suppression of its IL-18BP-mediated functional silencing. This would justify the poor anti-inflammatory role of skin in the determinism of low levels of free IL-18. It further suggests an age-related upregulation of IL-18BP in other anatomical districts in healthy centenarians or the involvement of other pro-IL-18BP cytokines in the skin. Contrary to IL-27, these cytokines serve as elixirs of life [27]. However, it should be pointed out that, to date, there is no confirmed chronological parallelism between immunosenescence and skin senescence. Nevertheless, a correlation has emerged between the cell cycle control protein p16INK4a, which is commonly accepted as a marker of cellular senescence, and markers of CD4+ T-cell immunosenescence [28]. Furthermore, there would seem to be an “immunity” from immunosenescence by certain subpopulations of skin-homing T cells, explaining the lack of clinical worsening of chronic T-cell-mediated inflammatory skin diseases in the elderly [29]. On the other hand, immunosenescence, accomplished by massive and repetitive antigen exposure over a lifetime, may be directly involved in the functional plasticity of skin CD4+ T cells, leading them to acquire a cytotoxic phenotype [30]. 

### 1.4. Epigenetic Influence on Skin Immunosenescence

Moreover, an increasingly investigated influence on skin immunosenescence occurs at the epigenetic level. The pioneering evidence on this issue came from the detection of a specific expression profile of microRNAs (miRNAs) in aged epidermal LCs compared with young LCs. This prompted the hypothesis of a direct engagement of aging-regulated miRNAs in LCs’ age-related functional and developmental changes, including the decline in tumor cell lysis and the impairment in antimicrobial function, primarily ensured by LCs in epidermal homeostasis conditions [31,32]. The most recent literature review on this topic provides a detailed insight into the different expression profiles of miRNAs between long-lived and younger individuals. It suggests centenarians’ involvement in inhibiting tumorigenesis and several age-related diseases. The influence of senescence-associated miRNAs in regulating the expression of various genes in skin cells has been emphasized, which can promote or decrease skin cell senescence. This is an interesting and perspective-filled area of research, as miRNAs are also expressed in other tissues and may play a role in organic aging [33]. Epigenetic influence also invokes new types of noncoding RNA, such as circular RNA (circRNA). In addition to its promising diagnostic, prognostic, and therapeutic role, especially in the oncologic field [34], circRNA has emerged as playing a key role in skin aging and immunosenescence, as well as in other age-related diseases, by regulating gene expression at the post-transcriptional level. CircRNA acts as an endogenous competitive antagonist in the binding of miRNAs to their target genes [35]. 

**Figure 1:**
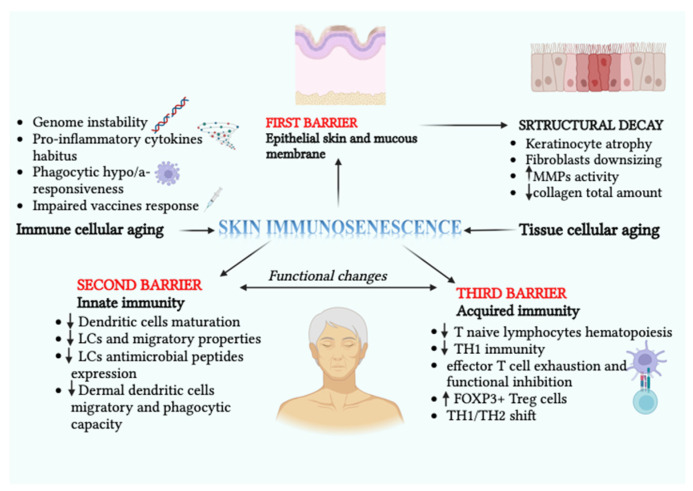
Schematic representation of the pathophysiological role played by immune cells and tissue aging in the setting up of skin immunosenescence. The involvement of all three immune skin barriers with their structural and functional changes is described. The latter concerns both the innate and acquired immunity domains, mostly engaging epidermal and dermal dendritic cells, in addition to the predominant involvement of the lymphocyte T compartment (T naïve, effector T cells). Created with BioRender.com.

Based on the current state of knowledge, the main objective of this review is to emphasize the role of cutaneous immunosenescence in the development of various dermatological disorders. Additionally, this review aims to shed light on the systemic pathological implications of immunosenescence and the underlying immunological mechanisms involved in the interplay between the skin and other organ-specific disorders. In this regard, we were moved to conduct a more in-depth evaluation of the immune interplay between the skin and other apparently estranged organs. This was prompted by the mounting recognition of an etiopathogenetic link between atopic dermatitis and cardiovascular as well as neuropsychiatric disorders [36,37,38,39,40,41,42,43]. Figure 1 represents the role of immune cells and tissue aging in the onset of skin immunosenescence.

## 2. Discussion

### 2.1. Skin Cancer and Immunosenescence

#### 2.1.1. Melanoma

In recent years, an ever-increasing body of evidence has demonstrated that immune system dysregulation can play a pivotal role in the physical degeneration processes and pathologic changes associated with aging. This suggests that immune system dysregulation could represent a potential target for the evaluation and management of elderly patients [44,45]. Senescence of the hematopoietic stem cell compartment, impairment of antigen-presenting cell functions, and T cell anti-tumoral potential have already been associated with aging. Immunosenescence may cause tissue and cell damage and immunosurvey escape, thus leading to tumor development [46]. Old age represents a serious risk factor for cancer development and is also associated with a poor prognosis [47]. In fact, cancer and aging can be considered two different manifestations of the same underlying process, in particular, the accumulation of cellular DNA damages [48,49]. The immune system has a dual role in cancer development. On the one hand, it can suppress tumor growth by destroying tumor cells, resulting in growth inhibition. On the other hand, it can promote tumor progression by selecting tumor cells best suited to survive in an immunocompetent host or by leading to the establishment of a tumor microenvironment that is (TME) favorable to tumor growth and metastasis [50,51,52]. Tumors are characterized by numerous somatic gene mutations and epigenetically dysregulated genes. The products of these genes are potentially recognizable as foreign antigens by the immune system, which represents the first line of defense [53]. However, cancer manages to avoid the immune system’s action through two main mechanisms: immune escape and immune tolerance [54,55]. Furthermore, the hypothesis is increasingly emerging that antitumor immunity may be compromised in the elderly due to a reduction in the number of naïve T cells, depletion of cancer-specific memory T cells, and the presence of high numbers of suppressor cells that instead inhibit the immune response [56]. In recent years, the treatment of advanced tumors, especially metastatic melanoma, has been revolutionized by the use of immune checkpoint inhibitors (ICIs), including PD-1/PD-L1 inhibitors and cytotoxic T-lymphocyte-associated antigen 4 (CTLA-4). These novel drugs have demonstrated promising results, offering new hope to cancer patients. On this topic, clinical trials have observed the impact of immunosenescence on ICIs effectiveness. Some tumors, such as head and neck and non-small-cell lung cancer, appeared to show a lower therapeutic response when they arose in elderly patients, while others, including melanomas, showed a similar response to ICIs therapy [57]. However, both retrospective studies and meta-analyses agree that age does not influence the response to immunotherapy since age-associated dysfunction of the immune system did not impact the ICIs’ effectiveness in aged patients [58,59]. Patients with stage IV melanoma, treated between 2012 and 2016 with ICIs were evaluated and divided into two groups older than 65 years and younger. The study elucidated that time to progression and overall survival were comparable between the two groups with no significant difference, supporting the hypothesis that aging does not appear to influence response to ICIs. From this evidence, it was deduced that elderly patients with metastatic melanoma should be treated in the same way as younger patients [59]. While ICIs have shown an increase in overall survival in patients with metastatic melanoma, the problem of the primary resistance of some tumors to these drugs has emerged. On this topic, Moreira et al. investigated whether senescence markers could predict the response to ICIs. They found that the loss of surface markers CD27 and CD28, or the expression of T-cell immunoglobulin and mucin domain 3 (Tim-3) and CD57 on T cells, was associated with resistance to checkpoint inhibitor blockade. These findings suggest that these phenotypes could serve as potential predictive biomarkers for ICI therapy [60]. Therefore, it would be useful to introduce panels for senescence markers to phenotype the lymphocytes before starting therapy with ICIs. This approach can help predict the patient’s response to therapy and select those who could benefit the most from this novel therapeutical choice.

#### 2.1.2. Cutaneous Squamous Cell Carcinoma

Immunosuppression is a well-established risk factor for the development of cutaneous squamous cell carcinoma (cSCC), a neoplasm caused by a malignant proliferation of keratinocytes originating from the epidermis and adnexal structures [61]. The immune mechanisms involved in cSCC development are complex [62]. On the one hand, reduced immunosurveillance/immunoediting, as well as immunosenescence, play a central role. On the other hand, the action of specific immune cells within the TME plays both an antitumor and pro-tumor dynamic role. Furthermore, a possible role in Cscc carcinogenesis has been attributed to oncogenic viruses. In this regard, the critical role of the immune system in the control of keratinocyte malignant degeneration emerged from the study on the differences in the risk of cSCC development between immunosuppressed and immunocompetent patients [63]. The role is further supported by the higher incidence and worse prognosis of cSCCs in organ transplant recipients (OTRs), caused by iatrogenic immunosuppression induced in these patients [64,65]. The incidence of cSCC in OTRs, which is up to 200 times that of the general population, closely correlates with the time elapsed since transplantation and, therefore, with the duration and intensity of immunosuppression. This correlation demonstrates a dose-dependent role in cSCC carcinogenesis and prognosis [65,66,67]. Patients with systemic disease-related immunosuppression, including chronic lymphocytic leukemia and non-Hodgkin’s lymphoma, also exhibit a moderately increased risk of cSCC, providing further evidence that immunosuppression drives carcinogenesis [68,69]. HIV-infected patients have a 2.6-fold increased risk of developing cSCC, which is inversely related to their CD4 count [70,71]. In an immunosuppressive setting, the impairment and changing of specific leucocytes in the periphery and in the TME, which influence immune functions, have been linked to enhanced cSCC malignancy [72,73,74]. In fact, reduced tumor-infiltrating cytotoxic CD8+ T cells and naive T lymphocytes in cSCCs of OTRs have been detected [75]. Low numbers of NK cells have been associated with increased cSCC risk in OTRs. On this topic, to identify patients at increased risk of developing cSCC, Bottomley et al. evaluated the risk of long-term morbidity in OTRs. Peripheral blood lymphocytes isolated from 117 stable transplant patients at high risk for SCC were analyzed. The results showed the strongest immunological predictor of future cSCC was that the percentage of CD8+ T cells expressing CD57, an immunosenescence marker. This was found to be strongly correlated with increased differentiation of CD8+ T cells. Furthermore, unlike NK cells, which can have their functionality modified by the continuous use of azathioprine, the CD57 phenotype is stable over time, even in the presence of already-developed cSCC. This finding could help identify OTRs who may benefit from more frequent and thorough dermatological screening, follow-up, and a reduction in immunosuppressive therapy whenever possible [76]. Moreover, the role of CD57+ senescent cells has been investigated in non-transplant populations and has been associated with a poorer prognosis [77,78,79]. Additionally, higher levels of CD57+ cells have also been correlated with the loss of CD4+ and CD8+ central memory T cells, another important source of antitumor immunity [80]. In fact, in established cSCC, tumor-infiltrating leucocytes, specifically CD4+ and cytotoxic CD8+ T cells, had a reduced density both in intra- and peritumoral tissues in immunosuppressed patients compared to healthy patients [72,81,82]. On the other hand, reflecting what has been observed in the periphery, there is an increase in Treg levels in the TME [83]. Moreover, the frequency of FOXP3+ Tregs, suppressors of antitumor response with the function of maintaining immunological tolerance to host tissues, in cSCC was strongly correlated with metastases and poorer clinical outcomes [84]. In a meta-analysis, Shang et al. evaluated the prognostic role of FOXP3+ Tregs in several tumors, demonstrating that FOXP3+ Treg infiltration had a negative effect on patient overall survival in most solid tumors, including melanoma [85]. From this, it can be deduced that Tregs may represent a new potential therapeutic target. Conversely, high Th1 serum levels and IFN-γ production, which represent the effector response against tumor antigens, have been associated with decreased susceptibility to cSCC development in immunosuppressed recipients [74]. Immunosuppression also increases the risk of viral proliferation in the skin compared to the general population, including HPV. Approximately 90% of cSCCs in OTRs contain HPV DNA, compared to 11–32% in immunocompetent skin samples [86]. The role of HPV as a cofactor in carcinogenesis has also been proposed, as it facilitates tumorigenesis by causing failure of DNA repair and apoptosis induced by UV-related damage [87]. The current data are inadequate to confirm the causal role of HPV since transcriptome analysis has failed to demonstrate the active replication of HPV within the cSCC [88].

**Figure 2:**
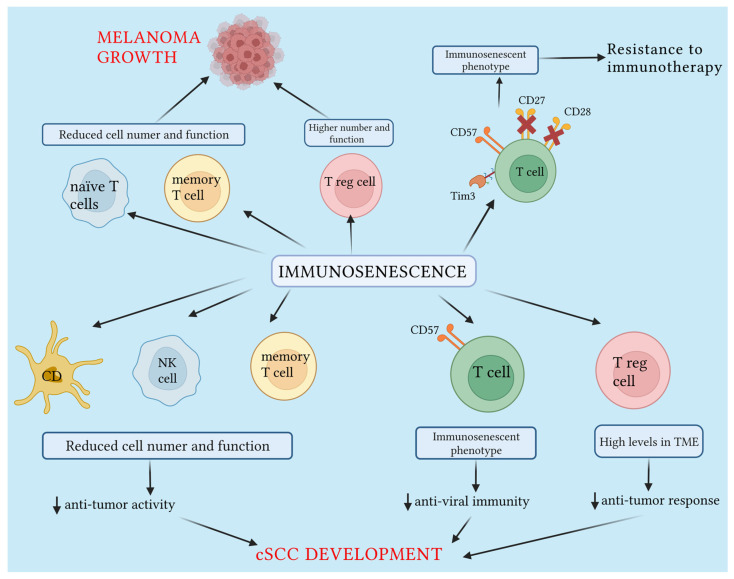
In melanoma, immunosenescence leads to reduced cell number and function of naïve T cells and memory T cells, with an increased number of Treg cells, thus inducing melanoma growth and metastatization process. Moreover, T cells express an immunosenescent phenotype characterized by reduced expression of CD27 and CD28 and higher expression of Tim-3 and CD57, a major marker of immunosenescence. This immunosenescent phenotype has been associated with resistance to immunotherapy treatment, suggesting a potential predictive biomarker of response to this novel therapy. In cSCC, immunosenescence is characterized by higher levels of CD57+ cells with a reduction of CD4+ and CD8+ central memory T cells, NK cells, and dendritic cells, while a higher level of Treg cells has been detected in the tumor microenvironment. This immunosenescent phenotype leads to reduced anti-tumor activity, reduced anti-viral immunity, and an anti-tumor response, which favors cSCC development in immunosenescent and immunosuppressed patients. Created with BioRender.com.

Switching to another skin cancer, Merkel cell carcinoma (MCC) is a neuroendocrine cancer that predominantly affects older people with impaired immune status. Its main causative agent is Merkel cell polyomavirus (MCPyV) [89]. Mazziotta et al. analyzed the sera of 226 elderly patients for anti-MCPyV IgGs. The results suggest that the virus circulates with a relatively higher prevalence in elderly people, while immunosenescence can explain the reduced IgG antibody response [90]. Dasanu et al., in a retrospective chart review, identified a cohort of immunocompromised patients with significant lymphopenia. They noted that MCC was more aggressive, with a time-to-death average of 290.1 days compared to 618.2 days in immunocompetent patients [91]. The available evidence suggests that immunosenescence and immune dysfunction may, at least in part, explain the higher prevalence and rapid increase in MCC cases in the elderly, particularly those aged 70 years and older. The role of immunosenescence in melanoma and cSCC development is represented in Figure 2. 

Highlights:

- Immunosenescence causes tissue and cell damage, and immunosurvey escapes, leading to cancer development.
- Cancer and aging can be considered two different manifestations of the same underlying process.
- Loss of antitumor immunity is characterized by naïve T cell reduction, depletion of cancer-specific memory T cells, and high numbers of suppressor cells.
- In melanoma, resistance to ICIs is characterized by the loss of CD27 and CD28 or the expression of Tim-3 and CD57 on T cells.
- Immunosuppression and immunosenescence are risk factors for cSCC development.
- Reduced tumor-infiltrating cytotoxic CD8+ T cells and naive T lymphocytes in the cSCC of OTRs have been detected, along with low numbers of NK cells.
- The percentage of CD8+ T cells expressing CD57 is the strongest immunological predictor of future SCC.
- In cSCC, tumor-infiltrating leucocytes, specifically CD4+ and cytotoxic CD8+ T cells, have reduced density both in intra- and peritumoral tissues in immunosuppressed patients, while Treg levels are increased in the TME.
- The frequency of FOXP3+ Tregs in cSCC is strongly correlated with metastases and poorer clinical outcomes, while high Th1 serum levels and IFN-γ production have been associated with decreased susceptibility to cSCC development.

### 2.2. Cutaneous Inflammatory Diseases and Immunosenescence

#### 2.2.1. Atopic Dermatitis

Atopic dermatitis (AD) is a chronic inflammatory skin disorder affecting up to 10% of adults, with an ever-increasing incidence in the elderly population due to society’s aging [92]. AD is a heterogeneous condition characterized by various phenotypes and endotypes based on the age of onset (pediatric vs. adult/elderly AD), ethnic origin (European-American AD vs. Asian AD), clinical features, and therapeutic response. AD endotype patterns include acute and chronic, as well as extrinsic (atopic-related) and intrinsic (nonallergic) AD [93,94,95]. Extrinsic AD represents about 80% of adult atopic patients and is associated with high serum levels of IgE. Elderly patients with this subtype show frequent allergic sensitization to airborne allergens, followed by food allergens. Intrinsic AD, depside being a less common subtype (≈20%), affects the elderly at a higher rate. It is characterized by normal or low serum IgE levels, the absence of an atopic background, and no sensitization to environmental allergens [94,95,96]. AD in elderly patients represents a newly defined subgroup whose etiology is complex and multifactorial. It is associated with a combination of environmental and genetic factors, immunosenescence phenomena, age-related epidermal barrier dysfunctions, cutaneous dysbiosis, functional impairment of sweat production, and external stimuli in the lifestyle [97,98]. There is a reciprocal relationship between skin barrier dysfunction, which is usually the primum movens, and the immune response, with elevated levels of inflammatory cytokines in aged skin [99]. In the acute phase of the disease, keratinocytes with damaged epidermal barriers produce cytokines known as “epithelial-derived cytokines,” including IL-33, IL-25, IL-18, and thymic stromal lymphopoietin (TSLP). These cytokines activate innate lymphoid cell type 2 (ILC2s) through specific receptors expressed on the membrane of ILC2s (IL-33R, IL-25R, IL-18R, and TSLPR) [100]. ILC2s recruit Th2 inflammatory cells, which in turn produce Th2 cytokines, IL-4 and IL-13, resulting in eosinophil recruitment [101,102,103,104]. ILC2s are also activated by IL-33, which stimulates basophils to produce IL-4, leading to IL-5 and IL-13 secretion [105,106]. Furthermore, IL-33 induces IL-31 release, a potent pruritic cytokine, whose levels strongly correlate with the severity of AD-associated symptomatology [107]. Hence, a self-perpetuating cycle is triggered: following itching, endogenous ligands of toll-like receptor (TLR)-4 stimulate the production of IL-23 by keratinocytes. This cytokine then activates dendritic cells (DCs) expressing the receptor IL-23R, which in turn triggers a Th22 aryl hydrocarbon receptor (AHR)-dependent immune response [108]. AHR activation stimulates robust expression of IL-22 on AD-affected keratinocytes. In fact, elderly skin shows greater Th22 polarization, which promotes epidermal hyperplasia and barrier defects and is thus presumably correlated with chronic immune stimulation over time [109]. In vitro studies have elucidated that IL-22 directly upregulates IL-33 and TSLP, further amplifying skin inflammation [110]. Furthermore, IL-4, IL-13, and IL-22, derived from Th2 and Th22, respectively, induce a reduction in stratum corneum thickness [111,112,113] and tight junction expression, thus inhibiting the production of defensive antimicrobial peptides in adult skin. This promotes superinfection by Staphylococcus aureus [114,115]. All of these factors act as drivers that can cooperate with others, leading to chronic inflammation and persistent disease in elderly patients [116,117].

**Figure 3:**
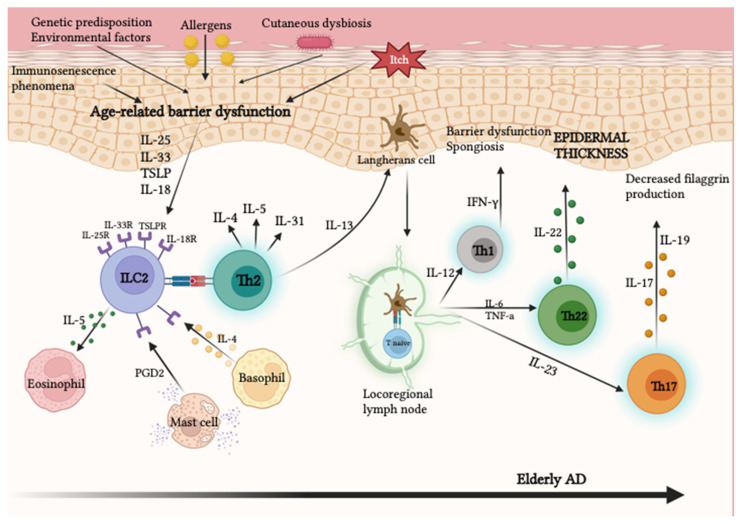
Following age-related skin barrier alteration, caused by immunosenescence phenomena, genetic and environmental factors, and cutaneous dysbiosis, damaged keratinocytes release epithelial-derived cytokines, including IL-25, IL-33, IL-18, and TSLP. ILC2s expressing the receptors for these cytokines (IL-25R, IL-33R, IL-18R, and STLP-R) on their surface are then activated, in turn releasing Th2 cytokines, including IL-5, IL-13, IL-4, and IL-31. Among these, IL-13 stimulates epidermal Langerhans cells, which in turn migrate to regional lymph nodes to prime naïve T cells by antigen presentation to promote the development of Th2 cells. In intrinsic AD and elderly patients, naïve T cells also differentiate into Th17, Th22, and Th1, respectively, through the stimulation of IL-23, IL-6, and IL-12. Th1 releases INF-γ which causes skin barrier dysfunction and spongiosis; Th17 produces IL-17 and IL-19, thus downregulating filaggrin production; and Th22 releases IL-22, which leads to the worsening of epithelial dysfunction and epidermal thickness. Created with BioRender.com.

Moving to the intrinsic phenotype, it is characterized by the lower expression of Th2-related cytokines (including IL-4, IL-5, and IL-13) and higher Th17 immune activation. In patients with intrinsic AD, Th17-related cytokines are highly expressed, both in acute and chronic diseases. Higher Th17 gene induction has also been detected in the AD skin of Asian patients. Although the exact role of IL-17 in AD is still unclear, some researchers propose that the Asian phenotype of AD may be an intermediate entity between the extrinsic European–American phenotype and psoriasis, where IL-17 has a known role in pathogenesis [118]. Asian AD shows significantly higher induction of Th17- and Th22-related cytokines (IL-17A, IL-19, and IL-22) and IL-17/IL-22-induced keratinocyte markers in lesional and/or nonlesional skin compared with those seen in European-American patients with AD [118]. Although Th2 axis activation is common across all AD phenotypes, intrinsic and Asian AD subsets show greater increases in Th17/Th22 activation. Activation of Th17 has been observed in older patients [119], who produce IL-17 [120]. This contributes to the onset of the barrier dysfunction typical of AD by reducing the expression of genes involved in cell adhesion and downregulating the production/degradation of filaggrin [121]. An increased level of Th1 signaling together with Th17 activation is also a possible immunological state for the intrinsic endotype of AD, which becomes more frequent among older patients. Chronic AD lesions in adults and the elderly show a conversion of polarization towards a Th1 phenotype [116]. This polarization is characterized by the production of IFN-γ and IL-12, which may play a role in the chronicity of inflammation and keratinocyte apoptosis [117]. Decreased Th2/Th22 activation and subsequent increased Th1/Th17 activation are peculiar immune characteristics of older atopic patients [93,95]. Hence, the severity of the clinical manifestations in the elderly patient since Th1 activation and increased IFN-γ production cause a reduction of Th2 proliferation and differentiation and immunoglobulin (Ig) E production, as well as IL-4 and its receptor, IL-4R [119]. The main immunological mechanisms of AD onset are described in Figure 3.

#### 2.2.2. Immunosenescence and Itching Diseases

Immunosenescence causes an increased predisposition to the development of itching in the elderly patient, which is often multifactorial and is also favored by a reduced function of the skin barrier as well as by neurological and psychological changes [122,123]. Chronic itching negatively impacts the quality of life and sleep. It can be attributed to several dermatoses, including seborrheic dermatitis and stasis dermatitis, as well as systemic conditions such as end-stage renal disease, diabetes, and blood disorders. Additionally, psychogenic conditions such as depression and anxiety can also cause chronic itching. Finally, even the use of many drugs can cause itching, regardless of the presence of a rash [124,125]. Xerosis is considered the most common cause of pruritus in elderly patients, with a prevalence ranging from 38 to 85% [126,127,128,129]. The alterations of the skin that lead to xerosis and itching are related to changes in the barrier function of the stratum corneum, pH variations, and reduced activity of the sebaceous and sweat glands. As far as the stratum corneum is concerned, this is the most superficial layer of the epidermis and has a barrier function capable of providing protection from external factors and preventing transepidermal water loss [130]. The stratum corneum is constantly subjected to cell turnover [131]. With skin aging, the normal cell turnover process can undergo functional alterations, resulting in the appearance of dry skin [131]. The intercellular matrix, composed of ceramides, cholesterol, and free fatty acids, also participates in the functions performed by the stratum corneum [132]. Reduced levels of ceramides have been found in geriatric patients, possibly due to reduced production of ceramide-generating enzymes [133]. Furthermore, a reduction in aquaporin 3, a protein that facilitates the transport of water and glycerol across the cell membrane to maintain epidermal hydration, was observed in dry skin as well as in aged skin [134,135]. Finally, the skin pH of the elderly tends to become more alkaline [136,137]. This favors the alteration of enzymatic activities in the stratum corneum, including enzymes that form ceramide and reduced lamellar body secretion [133,138,139,140]. Furthermore, alkaline pH increases the activity of serine proteases in the skin, leading to the activation of protease-activated receptor 2 (PAR2) receptors, which induces pruritus [141]. Therefore, changes in pH can induce or exacerbate chronic pruritus in the elderly population. Xu et al. conducted a study on elderly patients with chronic idiopathic pruritus and found an impaired immune response characterized by T- and B-cell lymphopenia, eosinophilia, and hypogammaglobulinemia. These findings suggest that immunosenescence may lead to a progressive reduction in the protective effects of Th1 cells, which favors a greater influence of Th2-driven allergic reactions. Additionally, immunosenescence may increase the susceptibility of the elderly to chronic itching [142].

Immunosenescence phenomena can also be associated with the development of autoimmune diseases, including pemphigus vulgaris, which is most prevalent in the elderly age. Studies have shown that in elderly patients, bullous pemphigoid can begin with itching as the only symptom in the absence of other specific manifestations, accompanied only by circulating autoantibodies. Typically, the onset of bullous diseases is characterized by the formation of blisters on the skin and mucous membranes [143]. From this, we deduce that it is important for the clinician to know this manifestation, to search for the autoantibody in the serum, and to start with the specific first-line therapy. The study by Wang et al. showed that senescent keratinocytes, induced by serial passaging or by oxidative stress, are more susceptible to pemphigus volgaris-IgG-mediated apoptotic death. This finding may explain the late onset of pemphigus vulgaris in old age. These cell subsets had significantly higher levels of senescence-associated b-galactosidase, p16, protein content per cell, and intrinsic fluorescence related to lipofuscin accumulation, while ki67, a marker of proliferation, was decreased [144]. Moreover, pemphigus vulgaris-IgG activates the extrinsic pathway of apoptosis, causing pemphigus to blister more easily in senescent cells. This is consistent with the fact that senescent keratinocytes are more sensitive to Fas-mediated apoptosis [145]. All this evidence can explain this disease’s increased frequency and severity in the elderly. 

#### 2.2.3. Psoriasis 

**Figure 4:**
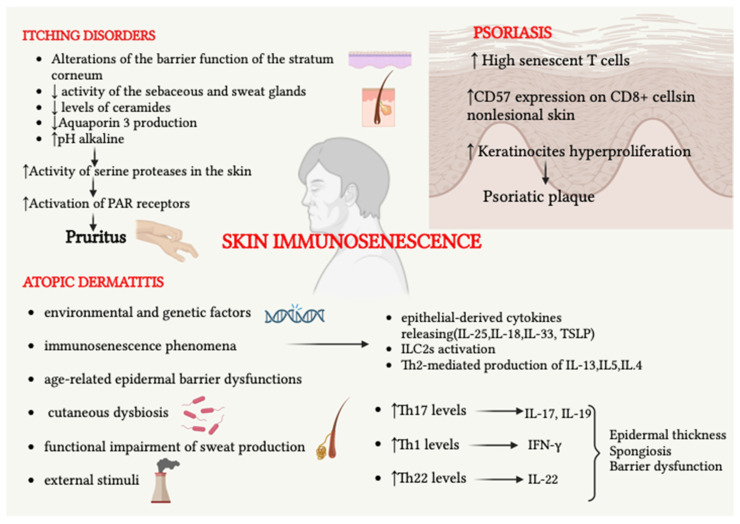
The role of skin immunosenescence in the onset of inflammatory cutaneous disorders is described. In senile pruritus, alteration of the barrier function of the stratum corneum, reduced activity of the sebaceous and sweat glands, reduced levels of ceramides, and an alkaline pH play a key role in the onset of the symptom. In psoriasis, higher levels of CD57 and CD8+ have been observed in nonlesional skin, since psoriasis is characterized by keratinocyte overproliferation. In AD, environmental and genetic factors, immunosenescent phenomena, age-related epidermal barrier dysfunctions, cutaneous dysbiosis, functional impairment of sweat production, and external stimuli represent the primum movens for the onset of the disease. Damaged keratinocytes release epithelial-derived cytokines, which in turn activate ILC2s to produce Th2-related cytokines including IL-13, IL-5, and IL-4. Moreover, in the elderly, elevated Th17, Th1, and Th22-related cytokines have been demonstrated, which favor the onset of epidermal thickness, spongiosis, and age-related barrier dysfunction.

Psoriasis is a chronically relapsing inflammatory skin disease with a bimodal distribution, with two peaks—early onset and late onset—occurring at 57 to 60 years [146]. The incidence of psoriasis after the age of 65 is progressively increasing due to the general aging of the population. Fortunately, the late onset of psoriasis is usually characterized by milder cutaneous manifestations compared with earlier-onset [147]. In current dermatological practice, about 10% of patients with psoriasis are elderly. Therefore, it is of fundamental importance to investigate psoriasis in this population. Elderly patients with psoriasis have a higher prevalence of various comorbidities, which presents a greater probability of adverse effects and impairment in quality of life [148,149]. As previously mentioned, the xerotic skin of the elderly tends to cause greater itching and consequent scratching, which can lead to the onset of psoriatic patches through the Koeber phenomenon. Šahmatova et al. highlighted that terminally differentiated or senescent T cells were present in higher proportions among CD8+ cells in patients with psoriasis. This proportion also correlated with disease duration, with higher levels observed in patients who had a history of psoriasis for more than 15 years. The authors concluded the study by asserting that T cells from patients with psoriasis reveal signs of excessive immune activation and features of early immunosenescence [150]. Moreover, Batista et al. studied the CD57 expression on CD8+ cells in the skin of patients affected by psoriasis. In fact, CD57 expression causes proliferative and survival inability, defining replicative senescence associated with conditions of chronic immune activation. The authors compared CD57 expression on lesional and nonlesional skin, thus showing that the frequency of CD57+CD4+ and CD57+CD8+ T cells was significantly higher in unaffected skin. This was explained by the fact that psoriasis is characterized by high cell turnover, whereby the T cells of the lesional skin show reduced expression of immunosenescence and replication inhibition markers, unlike what occurs in healthy non-lesional skin [151]. However, the major concern of clinicians in the elderly population affected by psoriasis regards the safety of the use of biological drugs, including new molecules that selectively target interleukins responsible for psoriatic disease. In fact, psoriasis represents a major risk factor for infectious diseases, which can be increased by administering immunosuppressive therapy, including methotrexate, cyclosporine, and biological drugs [152]. An evidence-based review suggests that the use of biological therapies may be associated with an increased risk of nonmelanoma skin cancer [153]. Since most psoriatic patients already have an increased risk of skin cancer due to a history of treatment with other therapies such as phototherapy or cyclosporine, biological therapies should be considered an additional risk factor for skin cancer. Evidence suggests that infection and severe adverse events are more common in the elderly population when compared to non-elderly adult patients. Therefore, patients with psoriasis are recommended to be carefully screened for possible latent systemic infections [154]. Hence, the need to ensure close follow-up of the elderly psoriatic patient through more frequent visits and routine screening for infectious diseases, including tuberculosis, hepatitis, and human immunodeficiency virus (HIV) infection. Figure 4 represents the main immunosenescent mechanisms in inflammatory diseases.

Highlights on cutaneous inflammatory diseases and immunosenescence

- AD in elderly patients represents a newly defined subgroup in which immunosenescence phenomena play a central role.
- Elderly skin shows greater Th22 polarization, which promotes epidermal hyperplasia and barrier defects.
- Elderly chronic AD lesions show a conversion of polarization towards a Th1 phenotype with the production of IFN-γ and IL-12.
- Immunosenescence causes an increased predisposition to the development of itching.
- Immunosenescence phenomena can also be associated with the development of autoimmune diseases, including pemphigus vulgaris and pemphigoid.
- Terminally differentiated or senescent T cells are present in higher proportions among CD8+ cells in patients with psoriasis.
- T cells of the lesional skin show reduced expression of immunosenescence and replication inhibition markers, unlike what occurs in healthy non-lesional skin. Psoriasis is characterized by a high cell turnover.
- Elderly psoriatic patients are recommended to be carefully screened for possible latent systemic infections before starting biological therapies.

### 2.3. Role of Immunosenescence in Cutaneous Infectious Diseases

The etiologic role of immunosenescence in promoting the onset of respiratory, gastrointestinal, skin, and soft tissue infections has been well-known for decades [155,156,157]. In addition to the recently explored unfavorable prognostic influence of immunosenescence on visceral leishmaniasis [158], a negative prognostic impact is also attributed to cutaneous leishmaniasis due to a likely common mechanism related to sestrins that drive immunosenescence of two immune cell types, namely NK cells and CD8+ T cells. These cell types were found to be more represented in the blood and skin lesions of elderly patients with cutaneous leishmaniasis compared to healthy controls of the same age [159]. More specifically, the intense proliferation of T cells would trigger their senescence and tendency to reside in the skin, resulting in the development of a SASP-like functional profile that is useful in controlling the parasite and influencing cutaneous immunopathology. This phenomenon has been strongly confirmed by extensive transcriptomic analysis [160,161] and is plausibly unconditioned by exhausted-like T cells [162]. Concerning viral infections, the etiopathogenetic role of immunosenescence has been primarily established for herpes viruses. In this regard, beyond its etiologic role in the occurrence of Epstein–Barr virus (EBV)-positive mucocutaneous ulcers [163], it has recently been investigated the synergistic action between immunosenescence and mycosis fungoides-related immunosuppressive status in the onset of HIV seronegative Kaposi’s sarcoma [164]. Moreover, immunosenescence itself would be enhanced by the action of cytomegalovirus, and both would synergistically be responsible for the reactivation of herpes simplex virus type 1 [165]. Immunosenescence would also appear to be able to both enhance the replicative efficacy of Varicella zoster virus in senescent skin fibroblasts and deter the host antiviral response [166]. Concerning bacterial infections, immunosenescence is considered a major risk factor for certain streptococcal infections [167]. However, this etiologic influence can be mitigated by the good maintenance of flagellin-dependent TLR5 signaling in senescent macrophages, which is subsidiary to caveolin-1’s immunoactivating action [168]. More recently, the etiopathogenetic influence of immunosenescence has also been recognized for active tuberculosis and leprosy with reference to monocyte phenotypic changes and age-related rearrangement of T lymphocyte subsets, respectively [169,170].

### 2.4. Cutaneous Microbiome as a Specific Pattern of Frailty 

As part of the skin microbiome, *Propionibacterium*, *Corynebacterium*, and *Staphylococcus* predominate. These are affected by changes in their composition that are site-dependent and secondary to age-related alterations in skin physiology as well as influenced by immunosenescence [22]. Furthermore, the skin microbiome can rightly be considered, similar to other microbiomes, a pro-inflammatory trigger due to an age-related imbalance between good and bad microbes, thus contributing to “inflammaging” [171]. Indirect confirmation of the microbiota’s immunomodulatory function comes from hypothesizing immune pathways through which prebiotics, probiotics, and postbiotics can influence immunogenicity and, consequently, effective vaccine response, especially in age groups including the elderly, for which it is of notable clinical relevance [172]. Given these premises, it is easy to understand the growing interest regarding the etiopathogenetic role of the microbiome, cutaneous, and others, in the occurrence of different skin diseases. The most recent body of evidence on the subject first recognizes the preponderant role of the skin microbiome as the primary “reservoir” of pathogenicity in older adults, especially referring to nosocomial strains and antimicrobial resistance genes. Such findings prompted Larson et al. to propose a specific pattern of frailty in the older adult: the Frailty-Associated Dysbiosis of the Skin (FADS), which would find its pathophysiological cornerstone in the age-related depletion of *Cutibacterium acnes* [173]. To delve into various dermatologic conditions, it is worth mentioning the increasing evidence of the etiopathogenic role of the skin microbiome in the observed transitional dysbiosis of progression from healthy skin to actinic keratosis to cSCC [174]. Additionally, recent research has shown that the oral microbiome may play a role in the onset of lichen planus by depleting species normally associated with oral health in favor of periodontitis-associated bacteria. Similarly, dysbiosis of both skin and gut microbiome would likely play an etiopathogenetic role in the onset of autoimmune bullous disorders [175]. On this subject, there is an interesting and topical line of research concerning the interaction between specific bioactive bacterial peptides, known as quorum-sensing peptides (QSPs), and host circulating immune cells. QSPs are thought to play an immunomodulatory role on immune cells in a pro-inflammatory way, thus promoting the onset of various diseases such as autoimmune disorders and cancer. The even more interesting aspect that emerged from the explorative data is that for certain of these peptides, the inflammatory effect would be age-dependent. Moreover, for a biologically relevant microbiome-host immune system interaction, a role of primary importance would hypothetically refer to QSPs produced by skin bacteria such as staphylococcal species [176].

### 2.5. Immunosenescence: A Subtle Hotline between the Skin and System-Specific Pathologies

**Figure 5:**
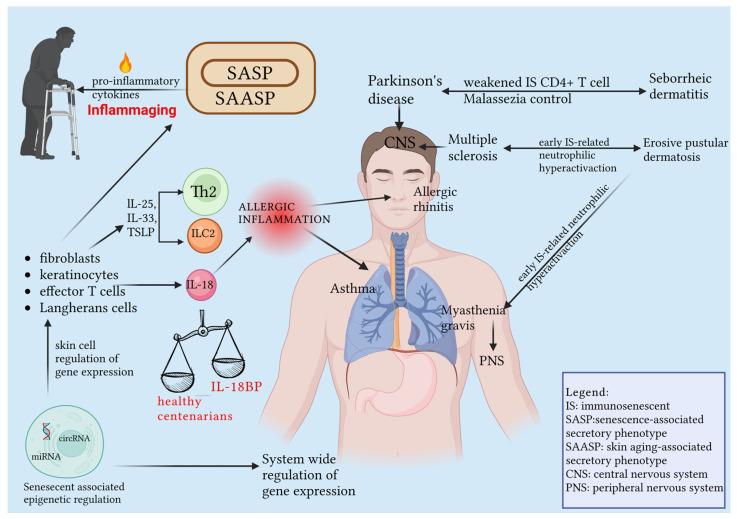
Schematic representation of skin senescent cells’ contribution to determining SAASP and thus SASP’s responsibility for the chronic low-grade inflammation that characterizes the elderly, known as inflammaging. Specifically, emphasized is the intriguing interplay between cutaneous and systemic immunosenescence and therefore the etiopathogenetic parallelism between various cutaneous and other organic pathologies (with a special emphasis on nervous and respiratory system disorders), including afferent immunological mechanisms underlying immunosenescence. Among the latter, a pivotal role is played by the prerogative of alarmin release by senescent skin immune and stromal cells, responsible for the genesis of Th2-mediated diseases and other autoimmune diseases, with the independent contribution of IL-18, a pro-inflammatory cytokine likely upregulated in the elderly with comorbidities but not in healthy centenarians due to the highly represented Il-18BP-mediated functional quenching action. Additionally, depicted is the epigenetic influence of senescence-associated miRNA and circRNA in the regulation of gene expression at the skin and systemic levels and consequently its influence on both skin aging, immunosenescence, and organismal aging due to a substantial number of genes influenced by such epigenetic regulation being shared between the skin and other organs. Created with BioRender.com.

After confirming the importance of accurately assessing the atopic status of geriatric patients with respiratory symptoms due to the socio-economic burden associated with the misdiagnosis of asthma and allergic rhinitis in this population, additional evidence supports the shift from Th1 to Th2 responses as the cause of allergic inflammation in older elderly [98,177,178]. More recently, Chen et al. have highlighted the potential causal role of skin immunosenescence in the onset of type 2 skin inflammation and related dermatologic conditions such as atopic dermatitis, chronic spontaneous urticaria, and bullous pemphigoid. They suggest that skin immunosenescence may lead to the development of a systemic skin-driven type 2 inflammation phenotype through the release of epithelial cell alarmin cytokines such as IL-25, IL-33, and TSLP from both the damaged epithelium and senescent skin stromal and immune cells. This may result in the upregulation of cytokines involved in allergic inflammation such as IL-4, -5, -9, 13, and -31 by ILC2 and Th2 cells [179]. In recent years, evidence of an intriguing etiologic link between cutaneous immunosenescence and its pathogenetic role in various dermatologic disorders and the systemic pathologic implications of immunosenescence, especially of the neurological matrix, has been gaining ground in the literature. In this regard, Laurence et al. justified the already known close association between Parkinson’s disease and seborrheic dermatitis by attributing a not negligible pathogenic role to T cell compartment immunosenescence, particularly involving the weakened control action of immunosenescent CD4+T cells on *Malassezia* overproliferation, both at skin and central nervous system sites [180]. Very recently, a well-founded hypothesis has emerged to further support the etiopathogenetic interplay involving cutaneous immunosenescence and systemic immunosenescence. It proposes that an aberrant release of neutrophil-stimulating cytokines and chemokines may result in putative neutrophilic hyperactivation, which serves as an etiologically common immunologic trigger for erosive pustular dermatosis of the scalp (EPDS) and two autoimmune neuropathies: multiple sclerosis and myasthenia gravis [181]. The interplay between cutaneous and systemic immunosenescence is represented in Figure 5.

## 3. Conclusions and Future Perspectives

Cellular senescence and its governing biomolecular mechanisms represent a matter of increasing scientific interest. This is due to their close correlation with the onset of several age-related diseases. This assumption would explain the increasing attention of research groups on an international scale to developing a new category of pharmaceuticals capable of targeting senescent cells, for this reason, labeled senotherapeutics, with the ultimate goal being the lengthening of health span [182]. In the context of such a vast new frontier of scientific research, special consideration is given to the aging processes concerning the immune cell compartment, specifically immunosenescence, with its related qualitative-quantitative changes. In our review, the focus is on the skin immunosenescence mechanisms, but it should not be considered a tight-compartment process. Skin immunosenescence is responsible for the onset of dermatological disorders of different matrices, but it also has fascinating system-wide implications that are still largely underexplored and fascinating. The outcome of this interplay between cutaneous and systemic immunosenescence could lead to direct etiopathogenetic parallelism between dermatological and various organic pathologies. Among these, respiratory and neurological diseases have emerged in the literature to date as potentially linked to skin immunosenescence. In the context of the immunological networking between the skin and other organs, future perspectives in this field should be directed towards an ever-better understanding of the mutual influence between the skin microbiome and host immunity, as well as the high interindividual variability that influences immunosenescence/inflammaging. This will lead to a more detailed “immunobiographic” definition of each individual, which can inform personalized approaches to disease prevention and treatment. At the same pace, research efforts in the field should tend towards an increasingly meticulous understanding of cytokine patterns involved in the skin-other organ pathogenetic shift. Moreover, a better characterization of binding proteins’ repertoire and their role in healthy centenarians should be pursued to find new molecules capable of serving as long-life elixirs. Last but not least would be to pursue a more in-depth elucidation of epigenetic regulatory mechanisms in cell aging involving miRNAs and circRNAs.

## Data Availability

Not applicable.
